# Life satisfaction and psychological wellbeing among medical students: the mediating role of psychological capital

**DOI:** 10.3389/fpsyg.2025.1614803

**Published:** 2025-10-10

**Authors:** Hanaa M. Abo Shereda, Rehab Alhazmi, Zeinab Kasemy, Eman Dawood, Evangelin Sally Jeya Singh, Iblouj Alkhalaf, Butool Alshehri, Taghreed Alanazi

**Affiliations:** ^1^Department of Psychiatric Mental Health Nursing, Faculty of Nursing, Menoufia University, Shibin El Kom, Egypt; ^2^College of Nursing, King Saud bin Abdulaziz University for Health Sciences, Riyadh, Saudi Arabia; ^3^King Abdullah International Medical Research Center (KAIMRC), Riyadh, Saudi Arabia; ^4^Department of Public Health and Community Medicine, Faculty of Medicine, Menoufia University, Shibin El Kom, Egypt; ^5^Faculty of Medicine, Menoufia National University, Tukh Tanbisha, Egypt

**Keywords:** life satisfaction, health professions students, mediating factor, psychological wellbeing, psychological capital

## Abstract

**Introduction:**

Health professions students are exposed to a range of academic, social, and emotional challenges, which have been found to affect their psychological well-being negatively.

**Objectives:**

This study aimed to assess psychological well-being, life satisfaction, and psychological capital levels among health professions students and the mediating role of psychological capital between psychological well-being and life satisfaction.

**Methods:**

A descriptive cross-sectional research design was employed using a sample of 217 health professions students from King Saud bin Abdulaziz University for Health Sciences. We utilized the Psychological Capital Questionnaire, Psychological Well-Being Scales, and the Satisfaction with Life Scale to collect data.

**Results:**

Findings revealed that psychological capital score had a positive and statistically significant impact on life satisfaction (Estimate = 0.23, 95% CI: 0.19 to 0.27, *p* < 0.001) and on psychological well-being (0.86, 95% CI: 0.70 to 1.03, *p* < 0.001), suggesting that psychological capital acted as a mediating factor in the relationship between psychological well-being and life satisfaction (*p* < 0.001).

**Conclusion:**

This study sheds light on the importance of cultivating psychological capital and its effect on satisfaction of life and psychological well-being in health professions students.

## Highlights

- Existing literature showed that Health professions students experience higher levels of psychological stress, anxiety, and depression compared to their age-matched peers who are not pursuing medical careers.
- Despite these challenges, health professions students exhibit moderate levels of psychological wellbeing and life satisfaction.
- Psychological capital serves as a mediator between psychological wellbeing and life satisfaction in health professions students, suggesting that fostering psychological capital is crucial for enhancing both wellbeing and life satisfaction in this population.

## Introduction

Health professions students are exposed to a range of academic, social, and emotional challenges, which have been found to affect their psychological wellbeing negatively (Hawsawi et al., 2025). Health professions students have to undergo a series of examinations during their training that are associated with emotional and professional demands. These include monthly tests, mid-semester tests, semester tests, term papers, internships, and oral exams. For this reason, health professions students experience heightened levels of psychological distress, including stress, anxiety, and depression than their age-matched peers who do not pursue a medical career (Nair et al., 2023). The majority of Saudi health professions students (97.1%) reported experiencing moderate to severe stress due to academic stressors such as exam frequency, curriculum demands, and performance pressures. Other regional studies corroborate these findings, with stress prevalence rates in Saudi medical students ranging from 53% to over 90%, depending on the institution and measurement tools used (Abd El Mawgod et al., 2024; Al-Shahrani et al., 2023; Alwhaibi et al., 2023).

Similar high stress rates have been reported in other countries—for example, medical students in Egypt (86.5%), Malaysia (41.9%), and Thailand (61.4%), as well as Iran (60.6%). Highlighting that academic stress among health and medical students is a worldwide matter that negatively affects their ability to succeed both academically and personally and poses high levels of threats to their physical and mental health, including life satisfaction and psychological wellbeing (Al-Shahrani et al., 2023; Mohammed et al., 2024; Ahmed and Quaderi, 2024; Palička et al., 2023; Nafees and Jahan, 2017; Borjalilu et al., 2015; Bhagat et al., 2018; Pastrana et al., 2022).

Psychological wellbeing is a broad concept that is variously influenced by physical health, mental state, social interactions, personal beliefs, and association with important characteristics of the environment (World Health Organization, 2004). The general definition of “psychological wellbeing” is a condition of “optimal psychological functioning” that includes six concepts: purpose of life (the degree to which participants feel purpose, meaning, and direction in their own lives); Autonomy (the extent to which they feel that they are acting in accordance with their personal beliefs); Personal growth (refers to participants’ abilities to utilize their unique talents and potential); Environmental mastery (the extent to which they master various situations in life); self-acceptance (refers to not only the understanding and acceptance of self but also the awareness of individual weak points and limitations); and positive relationships (the extent to which they feel deeply connected to others) (Ryff, 1989). People with high psychological wellbeing report feeling capable, comfortable, supported, and satisfied with their lives. Research suggests that psychological wellbeing plays a significant role in nursing students’ decisions to pursue a career in nursing and successfully adjust to life at college (Morales-Rodríguez et al., 2020).

Life satisfaction is an endorsement of a positive attitude towards one’s life in general (Hall, 2021). Life satisfaction is usually considered an important part of human wellbeing. In addition, life satisfaction assesses a person’s wellbeing by considering their mood, self-concept, and perceived ability to cope with life’s obstacles. Life satisfaction has been linked to aspects such as socioeconomic status, educational level, and experiences. In addition, people with high levels of life satisfaction have an advantage in terms of professional achievements, job performance, career happiness, and organizational commitment, all of which are linked to high levels of life satisfaction (Erdogan et al., 2012) Stressors such as academic pressure, time pressure, heavy workloads, strained relationships, inadequate funding and student supervision, fear of failure, and recurrent examinations are known to cause health professions students to experience a lack of life satisfaction (Abreu Alves et al., 2022).

Several studies have been conducted to investigate the correlation between psychological wellbeing and life satisfaction. One study examined the relationship between psychological wellbeing (life purpose, self-acceptance, environmental mastery, and positive relationships) and life satisfaction in young adults. The findings revealed that life satisfaction is related to psychological wellbeing in a positive way. Positive relationships and self-acceptance are more reliable indicators of meaning in life (Fatima et al., 2021). Compared to the numerous studies on risk factors for psychological wellbeing and life satisfaction, few have looked at protective variables.

Psychological resources have a significant impact on an individual’s perceived control over difficult situations (Seligman, 2004). Inadequate stress management can result in students becoming overwhelmed with their extensive academic commitments or continuing to feel like failures (Ling et al., 2014). Psychological capital is a significant psychological resource that researchers in organizational settings have focused on. Encouragingly, it has lately been expanded to other fields, like positive psychology in education, although it is still relatively new in academic medical education (Guerrero-Alcedo et al., 2022).

Psychological capital (PsyCap) refers to a set of resources that people can use to enhance their performance. In education, psychological capital refers to the identification and utilization of the best human abilities, which is aligned positively with psychological capabilities. It can be assessed, established, and accomplished efficiently to improve academic performance and outcomes, leading to an improvement in the overall wellbeing of students and teachers (Martínez et al., 2019).

Psychological capital (PsyCap) provides these resources: Hope, Efficacy, Resilience, and Optimism (HERO). For instance, a student with high PWB who faces a difficult exam may rely on their resilience (a PsyCap component) to bounce back from stress, their self-efficacy to prepare for the next challenge, their optimism to maintain a positive outlook, and hope to pursue future goals. In this model, PsyCap acts as the functional mechanism that translates the potential of high PWB into a sustained, positive judgment of life satisfaction, especially in the face of adversity (Luthans et al., 2007a; Luthans et al., 2007b; Luthans and YMCM, 2017).

Applying these concepts to the field of education, psychological capital can be said to enable optimal growth of individuals by giving them the confidence to make judgments and do the work necessary to complete academic duties (self-efficacy), proceed until they have achieved their educational objectives, or refocusing them to accomplish them (hope), making objective judgments about the academic events of today and tomorrow (optimism), and recovering from overwhelming situations or negative academic performance and emerging stronger (resilience) to achieve their academic goals (Martínez et al., 2021).

### The hypothesized model

**Figure 1:**
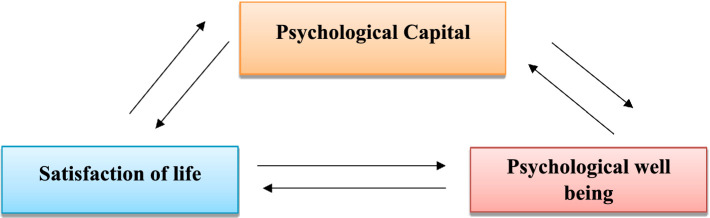
The hypothesized mediated model.

Based on the findings of previously mentioned studies, which demonstrated positive relationship between psychological wellbeing and life satisfaction from on one hand, and a positive relationship between psychological capital and wellbeing on other hand, we hypothesize that psychological capital may serve as a mediator factor in the relationship between psychological wellbeing and life satisfaction (Figure 1).

As far as we are aware, few studies have looked at the positive psychological aspects of academic students, including psychological wellbeing and life satisfaction. Despite growing interest in the psychological wellbeing of medical students, limited studies have explored the mediating role of psychological capital in the Saudi Arabian context, particularly across diverse health professions beyond medicine and nursing. This study addresses this gap and contributes region-specific insights into international literature. Therefore, the core purpose of this study was to assess the levels of satisfaction with life, psychological wellbeing, and psychological capital among health professions students in Riyadh, Saudi Arabia. Additionally, psychological capital was examined as a mediator between psychological wellbeing and life satisfaction.

## Methods

### Study design

An analytical cross-sectional research study examines the life satisfaction and psychological wellbeing of health professions students and assesses the effect of psychological capital on life satisfaction and psychological wellbeing.

### Setting

The study was carried out at the King Saud bin Abdulaziz University for Health Sciences in Riyadh. It is a government university in Saudi Arabia that has specialized in health sciences since 2005 and collaborates with the National Guard Hospital. It has its headquarters in Riyadh which has the main campus and two other campuses in Al-Ahsa and Jeddah. The University comprises seven colleges: College of Sciences and Health professions, Nursing college, College of Medicine, College of Pharmacy, College of Dentistry, College of Public Health and Health Informatics, and College of Applied Medical Sciences.

### Study participants

The study population included students at King Saud bin Abdulaziz University for Health Sciences in Riyadh, who met the following inclusion criteria; male and female students enrolled in one of the following colleges (College of Sciences and Health Professions, College of Nursing, College of Medicine, College of Pharmacy, College of Dentistry, College of Public Health and Health Informatics, and College of Applied Medical Sciences), aged from 18 to 25 years old. Students with known/ diagnosed medical or psychological disorders were excluded from the study.

### Sample size

**Figure 2:**
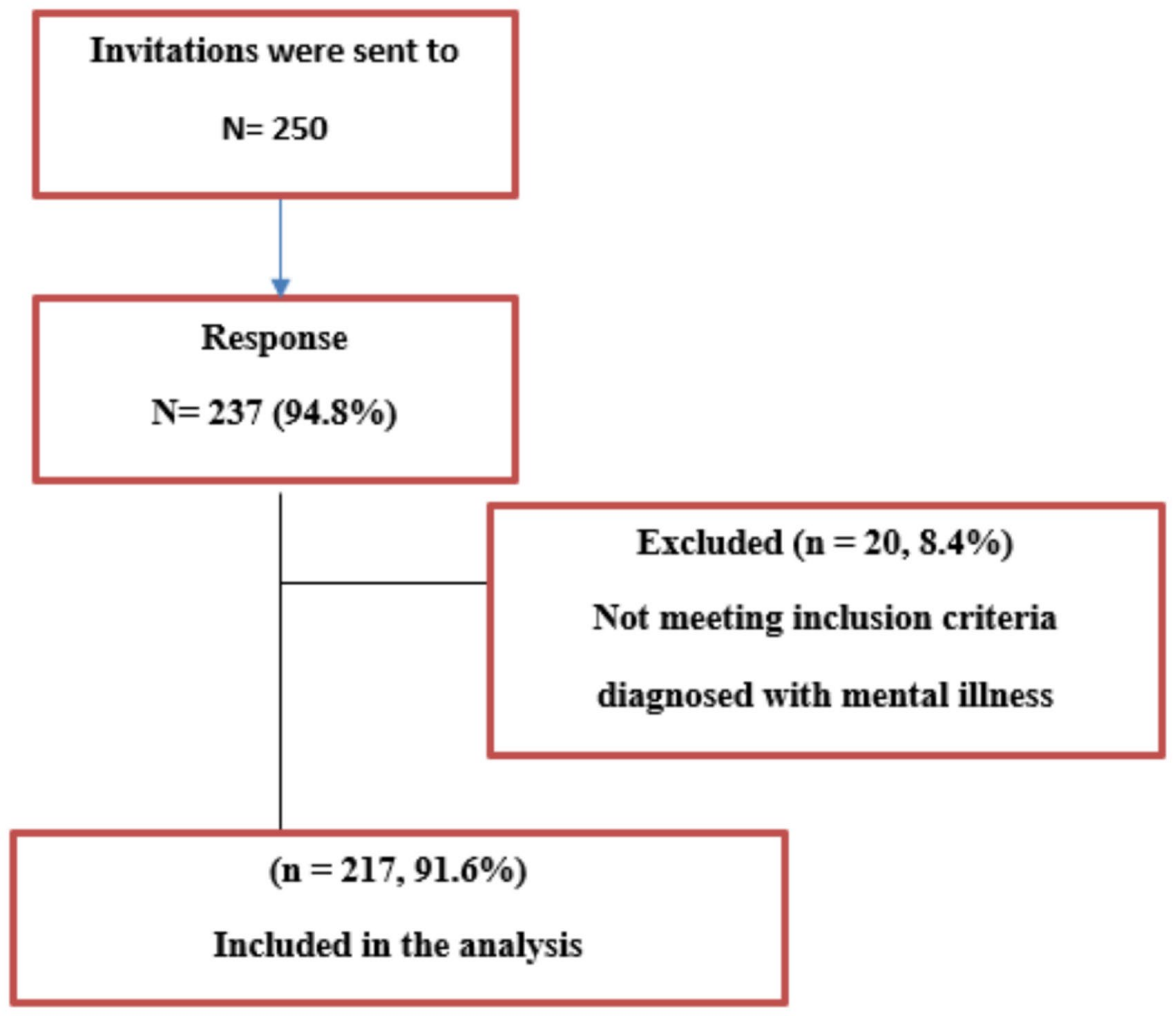
Flow chart of the studied participants.

Based on a study conducted in 2018, sample size had been calculated and it was estimated to be 183 students (Aboalshamat et al., 2018). Accounting for a dropout of 20% sample size had been increased to 230 students. Invitation was sent to 250 students of which A total of 237 students completed the study survey; 20 of them indicated that they had been diagnosed with mental illness, so they did not meet the inclusion criteria and were excluded from the analysis leaving a total of 217 students included in the analysis with a response rate of 94.8% (Figure 2).

### Data collection procedure

A convenience sampling technique was used after obtaining permission from the Ethical Committee at King Abdullah International Medical Research Center (KIAMRC), Riyadh, KSA. An online survey in Microsoft format was distributed to all health professions students through student board websites, social networking platforms such as WhatsApp, and in hard copy at colleges. Students were not allowed to respond to the questionnaire multiple times to prevent duplicate responses. Data collection started from the end of August 2023 to the end of December 2023.

### Data collection instruments

#### Demographic data

Demographic data involved age, gender, marital status, college, year of study, family members, parents’ occupation, family income, and history of diagnosed mental or physical illness.

### Satisfaction with life scale (SWLS)

The 5-item questionnaire was developed to measure people’s overall life satisfaction based on their cognitive judgments. It uses a 7-point rating scale, starting from 7 (strongly agree) and ending with 1 (strongly disagree), to express their level of agreement or disagreement with the five statements.

Total scores extended from 5:35. Lower values indicate higher levels of dissatisfaction: scores of 5 to 9 represent the highest level of dissatisfaction and scores 10: 14 imply dissatisfaction, substantial dissatisfaction is given for scores 15 to 19, 20 is neutral, slight satisfaction is interpreted for scores from 21 to 25, while 26 to 30 represent being satisfied, and 31: 35 indicate extreme satisfaction (Diener et al., 1985). Excellent test–retest reliability (*r* = 0.82) was reported for this scale, and an alpha of 0.87, indicating strong internal consistency. All scales used in the study were administered in their original English versions, as English is the official language of instruction at the university. All participants demonstrated proficiency in reading, writing, and understanding English; therefore, translation was not required.

### Psychological wellbeing scales (PWB)

The measure consists of 42 items assessing wellbeing across six sub-scales: positive relationships with others, self-acceptance, environmental mastery, autonomy, purpose in life, and personal growth. Each of these six subscales contains 7 items. These items include response categories based on a 7-point Likert scale, in which 1 indicates “strongly disagree” and 7 indicates “strongly agree.” According to Ryff’s (1989) original study, the six scales demonstrate satisfactory internal consistency (*α*) ranging from 0.86 to 0.93. These findings have been supported by subsequent research (Magyar-Moe, 2009; Magyar-Moe, et al., 2015). The total score was considered as the mean of the six subscales, where higher scores indicate higher levels of psychological wellbeing.

### Psychological capital questionnaire 24 items (PCQ 24 items)

The Psychological Capital Scale assesses an individual’s level of psychological capital. This scale is measured by 24 questions and consists of 4 sub-scale: optimism, self-efficacy, resilience, and hope. On a six-point Likert scale (where 1 = extremely disagree and 6 = extremely agree), participants specify how much they agree or disagree in response to all questions. Scores range between 24 and 144. The overall Cronbach’s α is 0.88/0.89. The overall reliability of the PsyCap scale consistently met conventional standards (Luthans et al., 2007a).

### Data analysis

The data was exported from Microsoft Forms and imported into R software version 4.2.2. Percentages and frequencies were used to display the categorical variables. The presentation of constant variables included standard deviations and mean values. Multiple linear regression analyses were used to determine the influence of psychological capital on life satisfaction and psychological wellbeing scores (both on the overall scale and the six subscales), while controlling for socio-demographic and academic characteristics of the participating students. Preacher & Hayes method was employed based on 1,000 bootstrap samples to estimate the average causal mediation effect, average direct effect, total effect, and proportion of the mediated effect of psychological capital in mediating the relationship between life satisfaction as an independent variable (X) and psychological wellbeing as a dependent variable (Y). Then the mediation analysis was reversed, with life satisfaction as the dependent variable (Y), psychological wellbeing as the independent variable (X), and psychological capital (M) as the mediator. A significant level of 0.050 was used in all analyses.

## Results

### Socio-demographic and academic characteristics

Table 1 provides an overview of the socio-demographic and academic criteria of the study participants, including 217 students who met the inclusion criteria. The gender distribution was relatively balanced, with 47.4% female and 52.5% male participants. The majority of participants were between 18 and 21 years old: 36.4% were 18–19 years old and 29.4% were 20–21 years old. The vast majority of participants were single (98.6%). Various academic disciplines were represented, with nursing (20.7%) and public health and health informatics (16.1%) being the most represented. The distribution across academic years reflects the different levels of education within the cohort. In terms of family characteristics, a significant proportion (54.8%) belonged to extended families. The participants also exhibited diversity in the employment status of their parents: 50.6% had a working father and 32.7% came from households where both parents were employed. The level of family income varied widely: 38.7% of participants reported an income of more than SAR 20.000, 37.3% were between SAR 11.000 and 20.000, 18.8% between SAR 5.000 and 10.000 and 05.1% had an income of less than SAR 5.000.

**Table 1:** Socio-demographic and academic characteristics of the study participants (*N* = 217).

Characteristic	(*N* = 217)	%
Gender		
Men	103	47.5
Women	114	52.4
Age		
18–19 years	79	36.4
20–21 years	64	29.4
22–23 years	62	28.6
24–25 years	12	5.5
Marital status		
Married	3	1.4
Single	214	98.6
Field of study		
Applied medical science	33	15.2
College of Science and Health Profession (COSHP)	36	16.6
Dentistry	23	10.6
Medicine	25	11.5
Nursing	45	20.7
Pharmacology	20	9.2
Public health and health informatics	35	16.1
Academic year		
First year	54	24.9
Second year	41	18.9
Third year	41	18.9
Fourth year	40	18.4
Fifth year	24	11.1
Sixth year	17	7.8
Family system		
Extended family	119	54.8
Nuclear family	98	45.2
Parents employment		
Employed father	110	50.6
Both are employed	71	32.7
Employed mother	7	3.2
Both are unemployed	29	13.4
Family income		
<5,000 SAR	11	5.1
5000–10000 SAR	41	18.8
11000-20000 SAR	81	37.3
>20,000 SAR	84	38.7

### Prevalence of satisfaction with life, psychological wellbeing, and psychological capital

As presented in Table 2, the mean score of SWLS was 22.73 ± 8.08, which suggested a moderate level of life satisfaction. Categorically, 65.4% of participants fell into the “satisfied” category, while 30.4% were categorized as “dissatisfied” and 04.1% as “neutral.” The overall mean of PWB was 188.32 ± 29.46, which suggests that individuals in the study experienced psychological wellbeing at a moderate level. The mean PCQ was 94.54 ± 22.23, suggesting a moderate level of psychological capital among the study participants. The mean self-perceived efficacy score was 24.42 ± 7.75, which suggests that, on average, participants have a moderate level of perceived efficacy in their professional roles. The mean hope score was 24.05 ± 7.25, suggesting that, on average, participants experience a moderate level of hope in their work-related endeavors. Participants had a mean score of 23.48 ± 5.43 on the resilience scale, indicating a moderate level of resilience on average. The optimism scale, with a mean of 22.60 ± 4.31, suggests a moderate level of optimism among the participants on average.

**Table 2:** Distribution of the studied participants regarding their score in satisfaction with life, psychological wellbeing, and psychological capital questionnaire.

Items	Mean ± SD/frequency (percentage%)
Satisfaction with life scale (SWLS)	22.73 ± 8.08
Satisfied	142 (65.44%)
Neutral	9 (4.15%)
Dissatisfied	66 (30.41%)
Psychological wellbeing (PWB)	188.32 ± 29.46
Environmental mastery	29.89 ± 5.09
Autonomy score	30.64 ± 6.07
Personal growth score	32.69 ± 6.42
Positive relations	31.94 ± 7.60
Purpose in life	31.92 ± 5.80
Psychological capital questionnaire (PCQ)	94.54 ± 22.23
Self-perceived efficacy	24.42 ± 7.75
Hope	24.05 ± 7.25
Resilience	23.48 ± 5.43
Optimism	22.60 ± 4.31

SWLS = Satisfaction with Life Scale; PWB = Psychological Wellbeing; PCQ: Psychological Capital Questionnaire; SD = standard deviation.

Table 3 shows the results of the multiple linear regression analysis, which examines the impact of psychological capital on life satisfaction, while controlling various socio-demographic and academic characteristics. The values of psychological capital had a positive and statistically significant effect (Estimated value = 0.23, 95.0% CI: 0.19 to 0.27, *p* < 0.001), indicating that an increase in Psychological Capital was associated with a positive increase in Satisfaction with Life scores. Among the socio-demographic and academic characteristics, family income was significantly associated with life satisfaction. In addition, the results of a multiple linear regression analysis examining the impact of psychological capital on the psychological wellbeing score, while controlling for various socio-demographic and academic characteristics shows that the psychological capital score had a substantial positive effect (0.86, 95.0% CI: 0.70 to 1.03, *p* < 0.001), indicating that an increase in psychological capital was associated with a significant positive increase in psychological wellbeing scores. The marital status of being single emerged as a statistically significant positive predictor of psychological wellbeing (Estimate 29.41, SE 14.62, 95.0% CI: 0.58 to 58.25, *p* = 0.046).

**Table 3:** Multiple linear regression for the effect of psychological capital on satisfaction with life score and psychological wellbeing score considering the socio-demographic and academic characteristics.

Predictors	Estimate	95% CI	*p*	Estimate	95% CI	*p*
(Intercept)	−2.26	(−11.6, 7.14)	0.636	80.77	**(45.4, 116.10)**	<0.001*
Psychological capital score	0.23	(0.19, 0.27)	<0.001*	0.86	(0.70, 1.03)	<0.001*
Age						
18–19 years (ref)						0.393
20–21 years	1.63	(−1.75, 5.01)	0.342	−5.51	(−18.20, 7.18)	0.89
22–23 years	0.49	(−3.67, 4.65)	0.816	−1.1	(−16.72, 14.52)	0.425
24–25 years	1.03	(−4.13, 6.19)	0.694	−7.85	(−27.22, 11.51)	
Sex						
Female (ref)						
Male	1.31	(−0.73, 3.35)	0.207	−1.23	(−8.89, 6.43)	0.753
Marital status						
Married (ref)						
Single	−2.95	(−10.63, 4.73)	0.449	29.41	(0.58, 58.25)	0.046*
Field of study						
Nursing (ref)						
Applied medical science	−2.5	(−6.08, 1.09)	0.171	−2.26	(−15.72, 11.21)	0.741
College of Science and Health Profession	−1.26	(−4.78, 2.26)	0.481	−2.56	(−15.78, 10.67)	0.703
Dentistry	−1.84	(−6.15, 2.46)	0.4	1.77	(−14.39, 17.94)	0.829
Medicine	−1.72	(−5.53, 2.08)	0.373	0.68	(−13.61, 14.98)	0.925
Pharmacology	−1.9	(−5.79, 1.99)	0.337	−2.83	(−17.44, 11.79)	0.703
Public health and health informatics	1.46	(−1.95, 4.87)	0.399	7.96	(−4.84, 20.75)	0.222
Academic year						
First year (ref)						
Second year	0.04	(−2.80, 2.89)	0.976	1.59	(−9.09, 12.27)	0.769
Third year	−0.58	(−4.49, 3.33)	0.769	−0.26	(−14.95, 14.42)	0.972
Fourth year	−0.04	(−4.08, 4.00)	0.983	3.43	(−11.74, 18.60)	0.656
Fifth year	−0.01	(−5.02, 4.99)	0.995	7.73	(−11.07, 26.53)	0.418
Sixth year	−0.01	(−5.79, 5.76)	0.997	−9.52	(−31.21, 12.18)	0.388
Family system						
Extended family (ref)						
Nuclear family	1.08	(−0.78, 2.93)	0.253	3.58	(−3.38, 10.54)	311
Parents’ employment						
Employed father (ref)						
Employed mother	−0.15	(−5.01, 4.71)	0.952	−0.93	(−19.19, 17.33)	0.92
Both are employed	−0.13	(−2.07, 1.80)	0.892	−3.77	(−11.04, 3.49)	0.307
Both are unemployed	0.61	(−2.09, 3.31)	0.655	−5.99	(−16.14, 4.15)	0.245
Family income						
<5,000 SAR (ref)						
5,000–10,000 SAR	5.17	**(0.92, 9.42)**	0.017*	−0.08	(−16.05, 15.89)	0.992
11,000–20,000 SAR	5.87	**(1.68, 10.06)**	0.006*	−4.31	(−20.05, 11.43)	0.59
>20,000 SAR	5.34	**(1.13, 9.56)**	0.013*	1.02	(−14.82, 16.86)	0.899

N.B. Results are derived from multiple linear regression analyses. Reference categories for categorical predictors were Age (18–19 years), Sex (Female), Marital status (Married), Field of study (Nursing), Academic year (First year), Family system (Extended family), and Parents’ employment (Father employed). *Statistically significant at *p* < 0.05. The model explained 49% of the variance in Satisfaction with Life Score (*R*^2^ = 0.49) and 46% of the variance in Psychological Wellbeing Score (*R*^2^ = 0.46). Bold values mean significant *p* values.

The first part of Table 4 presents the results of a mediation analysis conducted using the Preacher & Hayes method examining the role of psychological capital in mediating the relationship between life satisfaction as an independent variable (X) and psychological wellbeing as a dependent variable (Y). The results show that the average causal mediation effect was significant (*p* < 0.001) while the direct effect was non-significant, indicating that psychological capital mediated the relationship between life satisfaction and psychological wellbeing. Additionally, the proportion of the mediated effect was substantial (74.0%), suggesting that a considerable portion of the total effect is explained by the indirect pathway through psychological capital.

**Table 4:** Mediation analysis for psychological capital on the effect of life satisfaction on psychological wellbeing.

Effects	*ß*	95% CI		*p*
		Lower	Upper	
Average causal mediation effect	1.3	0.92	1.74	<0.001*
Average direct effect	0.45	−0.04	0.94	0.072
Total effect	1.76	1.31	2.21	<0.001*
Proportion of mediated effect	0.74	0.53	1.03	<0.001*

Mediation analysis for psychological capital on the effect of psychological wellbeing on life satisfaction using				
Effects	*ß*	95% CI		*p*
		Lower	Upper	
Average causal mediation effect	0.1	0.07	0.13	<0.001*
Average direct effect	0.03	0	0.06	0.06
Total effect	0.13	0.1	0.16	<0.001*
Proportion of mediated effect	0.76	0.55	1.02	<0.001*

β: regression coefficient; CI = confidence interval; *p < 0.050.

In the second part of Table 4, the mediation analysis was reversed, with life satisfaction as the dependent variable (Y), psychological wellbeing as the independent variable (X), and psychological capital (M) as the mediator. The results show that the average causal mediation effect was significant, indicating that psychological capital mediated the relationship between psychological wellbeing and life satisfaction (*p* < 0.001). Moreover, a substantial proportion of the total effect was explained by the indirect pathway through psychological capital (76.0%).

The result of the effect of socio-demographic and academic factors on psychological capital shows that the field of study exhibited significant effects, with students in Applied Medical Sciences (Estimate: 20.21, 95.0% CI: 8.77 to 31.64, *p* = 0.001), Science and Health Professions (Estimate: 28.12, 95.0% CI: 17.25 to 38.99, *p* < 0.001), Dentistry (Estimate: 17.36, 95.0% CI: 3.42 to 31.31, *p* = 0.015), Medicine (Estimate: 23.58, 95.0% CI: 11.52 to 35.64, *p* < 0.001), Pharmacy (Estimate: 17.65, 95.0% CI: 5.10 to 30.20, *p* = 0.006), and Public Health and Health Informatics (Estimate: 20.85, 95.0% CI: 10.05 to 31.66, *p* < 0.001) exhibiting higher social capital compared to those in Nursing. Additionally, the family system significantly influenced psychological capital. Individuals from nuclear families reported lower psychological capital compared to those from extended families (Estimate: −6.85, 95.0% CI: −12.87 to −0.83, *p* = 0.026).

## Discussion

Research on students’ mental health has recently gained attention, and few studies have investigated the psychological wellbeing and life satisfaction of students, especially health profession students in Saudi Arabia and other countries (Franzen et al., 2021). Therefore, the goal of this study was to investigate the levels of life satisfaction and psychological wellbeing of students in health professions. It also aimed to assess the impact of psychological capital on both life satisfaction and psychological wellbeing.

Regarding the life satisfaction of students in health professions, the results of the present study show that the medical profession students had a moderate level of life satisfaction. Accordingly, the majority of participating medical profession students were positive about various aspects of life satisfaction. As more than half of the students indicated that their lives were close to their ideal, and that their living conditions were excellent Although health professions students face many challenges, such as long training periods, emotional stress related to hospital training, problems with patients, long contact hours, and high completion, it is possible that the students in our sample reported moderate levels of satisfaction, which could be because they were accepted to one of the health professions colleges that have the highest admission requirements. Moreover, most health professions students often perceive their chosen field as offering social prestige, economic stability, and meaning in life; therefore, they felt that their lives were close to what they planned. These findings are similar to those in other Saudi Arabian studies that found that approximately two-thirds of the participants were satisfied with their lives (Aboalshamat et al., 2018). Similarly, studies in India and China reported high levels of satisfaction among participants (Lacey et al., 2000; Shi et al., 2015; Liang et al., 2025). It has also been clarified that a meaningful life and positive future expectations are linked with personal satisfaction, psychological wellbeing, and positive affect, which are crucial for life satisfaction (Saunders et al., 2023).

Regarding the psychological wellbeing (PWB) of the health professions students, the mean of the total score indicated that the participating students experienced moderate psychological wellbeing. Similarly, students reported moderate levels across the six psychological wellbeing subscales. Although it is well documented in the literature that health professions students, in particular, face challenges to their mental health and wellbeing, it was expected that they would report low levels of psychological wellbeing. Unexpectedly, we found that the majority have moderate levels of psychological wellbeing, which is attributed to the university’s efforts to provide quality education that addresses student wellbeing through various academic support programs (academic advising), extracurricular activities, and student wellness centers. In addition, they offer many recreational activities through the student club and involve students in real life by encouraging them to participate in community activities (Peleias et al., 2017; Dyrbye et al., 2019; Martins et al., 2017). Another important explanation could be the positive relationship between life satisfaction and psychological wellbeing. As health professions students are satisfied with their lives and career choices, accepting all these circumstances and recognizing them as challenges to be overcome, they can cope with these demands and achieve a moderate level of psychological wellbeing (Yeşi̇ltepe et al., 2022).

This finding is consistent with Alshehri (2021), who reported that their participants generally had an average level of mental wellbeing. Regarding the correlation between the socio-demographic data of participating students and their psychological wellbeing, the psychological wellbeing of the participating students did not differ significantly based on their gender (Alshehri, 2021). Parallel to these findings, Morales-Rodríguez et al. (2020) reported that sex had no statistically significant variation in psychological wellbeing in their study.

Concerning the correlation between the type of college and psychological wellbeing, there was no significant relationship. On the other hand, findings from previous studies were controversial, as Morales-Rodríguez et al. (2020) found that psychological wellbeing dimensions’ scores had statistically significant differences (purpose-in-life and environmental mastery) concerning the type of course /college. Additionally, Alshehri (2021), revealed that high mental wellbeing was found among College of Pharmacy students and that students from the College of Nursing were the lowest (Alshehri, 2021). In contrast, Franzen et al. (2021) concluded that studying pharmaceutical sciences and psychology was associated with decreased psychological wellbeing and increased stress. Even though the age of participants was not statistically significant for psychological wellbeing, Franzen et al. (2021) reported that increased age, even within young participants, was associated with greater psychological wellbeing.

As far as we know, the current study is the first of its kind in Saudi Arabia to assess the level of psychological capital of college students and its moderating role on life satisfaction and psychological wellbeing of health professions students. In reference to the level of psychological capital, our findings show that the mean score of the total psychological capital questionnaire indicated a moderate level of psychological capital on average among the participating students. On top of that, participating students showed a generally positive outlook on the four domains of Psych Cap, which are considered crucial factors that should be present in almost all students who are looking forward to working in the health professions field. Since self-perceived efficacy in various work-related scenarios is necessary for professionals who continuously face challenging, different, and complicated situations every single day that require not only self-perceived efficiency but also hope and optimistic attitudes. Conjointly, the majority of the participating students expressed optimism and confidence in their ability to navigate challenges and think of lots of ways around any problem to achieve their work-related goals. Likewise, they reported that they expect the best when faced with uncertainty at work, consider the positive side of things, and express optimism about their future work-related experiences.

Consistently, Rezaei et al. (2018), in their study at “Kurdistan University of Medical Sciences”, found that psychological capital’s mean score was 89.09 ± 9.98 Rezaei et al. (2018). In analyzing the connection between psychological capital and socio-demographic data, our results indicated no significant relationship between the two. Gong et al. (2018), supported our findings regarding sex but disagreed regarding socioeconomic status. They found no observable differences between women and men concerning psychological capital, pleasant emotion, and study engagement (Gong et al., 2018). However, they did identify a significant difference in psychological capital based on family economic level. A statistically significant association was found among the type of college, gender, faculty place, academic degree, and psychological capital, which was reported to be effective in enhancing students’ psychological capital (Rezaei et al., 2018).

Regarding the effects of psychological capital on life satisfaction, multiple linear regression analysis showed that the scores of psychological capital questionnaire had a positive and statistically significant effect, suggesting that an increase in psychological capital correlates with a positive increase in life satisfaction scores. Accordingly, Wang et al. (2022) found that PsyCap and its subscales were significantly positively associated with life satisfaction (Avey et al., 2011). These results are particularly confirmed by Luthans et al. (2007a), who found in early scientific literature that psychological capital, when assessed universally, is a reliable indicator of performance and satisfaction.

As regards psychological capital in mediating the relationship between life satisfaction as an independent variable (X) and psychological wellbeing as a dependent variable (Y) and then, the mediation analysis was reversed, with life satisfaction as the dependent variable (Y), psychological wellbeing as the independent variable (X), and psychological capital (M) as the mediator. The result showed that the direct impact of life satisfaction was no longer significant when psychological capital was included, indicating that the relationship between life satisfaction and psychological wellbeing was partially mediated by psychological capital. Similarly, the reverse path means that PsyCap partially mediated the correlation between psychological wellbeing and life satisfaction. Moreover, the proportion of the mediated effect was substantial for both pathways, suggesting that a substantial part of the overall effect is explained by the indirect pathway through psychological capital. Although, to our knowledge, no published study has investigated psychological capital as a mediator between life satisfaction and psychological wellbeing, some researchers have examined the mediating role of PsyCap between life satisfaction and other factors such as depression and anxiety. They found that PsyCap mediates the correlation between life satisfaction on one side and depression and anxiety (Wang et al., 2022). Additionally, other studies revealed that the association between mental health and spiritual wellbeing is partially mediated by PsyCap (Parviniannasab et al., 2022). Moreover, Chaffin et al. (2023) stated that PsyCap has a significant mediating role between satisfaction/performance and LGO.

## Limitations of the study

The limitations of this study include, first, using a self-reported method in gathering data can decrease the precision of the collected data. Secondly, utilizing a convenience sample technique restricts the generalizability of results. The adopted cross-sectional design does not permit the determination of causal relationships. For this reason, additional longitudinal studies are needed to evaluate the relationships among psychological wellbeing, life satisfaction, and psychological capital. Furthermore, other factors that may be influenced by psychological capital, particularly negative psychological factors such as burnout, anxiety, and stress, need to be considered, as this study focused solely on positive factors, which are psychological wellbeing and life satisfaction. Finally, exclusive reliance on quantitative methods may limit the depth of insight into students’ personal experiences. Future research could benefit from qualitative or mixed-method approaches.

## Conclusion

The current study found that health professions students had moderate levels of satisfaction with life, psychological wellbeing, and psychological capital. Higher family income was correlated with higher scores of life satisfaction in relation to psychological capital. Moreover, single students, compared to married students, tended to have greater degrees of psychological wellbeing. Furthermore, the family system significantly influences psychological capital, with individuals from nuclear families having lower psychological capital compared to those from extended families. Notably, the field of study had a substantial impact, with students in applied medical science, dentistry, medicine, pharmacology, public health, and health informatics showing higher psychological capital than nursing students do. Additionally, findings indicate that psychological capital acts as a mediating factor in the association between psychological wellbeing and satisfaction with life and vice versa. By cultivating these positive psychological resources, educational institutions can play a critical role in reducing the stress and challenges associated with medical education, leading to improvements in the health professions students’ psychological wellbeing and overall satisfaction with life.

### Theoretical and practical implications

Theoretically, the findings of this study reinforce the importance of positive psychological constructs, particularly psychological capital, in research on wellbeing and life satisfaction. Practically, medical education institutions and educators should provide support for students with low psychological wellbeing and life satisfaction, considering their social and personal factors. Additionally, stakeholders should design, implement, and evaluate targeted interventions and programs aimed at cultivating psychological capital resources to enhance students’ psychological wellbeing and life satisfaction. Furthermore, further longitudinal studies are needed to examine the long-term effect of psychological capital intervention on students’ life satisfaction and psychological wellbeing.

## Data Availability

The original contributions presented in the study are included in the article/supplementary material, further inquiries can be directed to the corresponding author/s.
